# Association Between Fibromyalgia and Risk of Developing Sarcopenia According SARC-F: A Cross-Sectional Study

**DOI:** 10.3390/diagnostics16010062

**Published:** 2025-12-24

**Authors:** Blanca Pedauyé-Rueda, Eduardo Cimadevilla Fernández-Pola, Hilin Hussein, Cristina Ojedo-Martín, María Jesús Fernández-Aceñero, Juan Hernández-Lougedo, Noemí Mayoral-Gonzalo, Juan Pablo Hervás-Pérez, Edurne Úbeda-D’Ocasar

**Affiliations:** 1 Physiotherapy and Health Research Group (FYSA), Department of Physiotherapy, HM Faculty of Health Sciences, Camilo José Cela University, 28692 Madrid, Spain; blanca.pedauye@ucjc.edu (B.P.-R.); ecimadevilla@ucjc.edu (E.C.F.-P.); husseinhilin01@gmail.com (H.H.); cojedo@ucjc.edu (C.O.-M.); jlougedo@ucjc.edu (J.H.-L.); eubeda@ucjc.edu (E.Ú.-D.); 2 HM Hospitals Health Research Institute, 28015 Madrid, Spain; 3 Department of Legal Medicine, Psychiatry and Pathology, Faculty of Medicine, Complutense University of Madrid, Plaza Ramón y Cajal s/n, Ciudad Universitaria, 28040 Madrid, Spain; jmariajesus.fernandez@salud.madrid.org; 4 Department of Surgical Pathology, Hospital Clínico San Carlos, 28040 Madrid, Spain; 5 Analytical Chemistry Unit, Department of Chemistry in Pharmaceutical Sciences, Faculty of Pharmacy, Complutense University of Madrid, Plaza Ramón y Cajal s/n, Ciudad Universitaria, 28040 Madrid, Spain; jphervas@ucm.es

**Keywords:** fibromyalgia, sarcopenia, musculoskeletal disorder, pain, anxiety and depression

## Abstract

**Background:** Fibromyalgia (FM) is a condition characterised by chronic pain, which may or may not be associated with muscular stiffness. Sarcopenia is the progressive loss of muscle mass and strength. The loss of muscle mass is a key factor in the progression of both fibromyalgia and sarcopenia and therefore warrants thorough evaluation. It has been demonstrated that obesity directly influences factors that increase pain perception and disease severity and reduce quality of life. The primary objective of this study was to examine the association between fibromyalgia and the increased risk of developing sarcopenia. **Methods**: The sample consisted of 84 patients diagnosed with FM. We assessed sociodemographic characteristics, anthropometric variables (circumferential and ultrasound) pain with a Visual Analogue Scale (VAS) and algometry, risk of developing sarcopenia with SARC-F, quality of sleep, anxiety, and depression using validated questionnaires. **Results:** A total of 96.3% of the participants were women. Overall, 56.3% of the sample presented a high risk of sarcopenia according to SARC-F, VAS scores showed significant negative correlations with anxiety (*p* < 0.01) and with almost all algometric measures (*p* < 0.05). The Fibromyalgia Impact Questionnaire (FIQ) demonstrated a positive and significant correlation with sleep quality (*p* < 0.01) and depression (*p* < 0.01). Furthermore, presence of a high risk of sarcopenia according to SARC-F was significantly associated with FIQ scores (*p* = 0.002) and depression (*p* < 0.001). **Conclusions:** There is a significant association between the impact of FM and a high risk of developing sarcopenia according to SARC-F. This population exhibits a high degree of pain, which are significantly associated with elevated levels of anxiety and depression.

## 1. Introduction

Fibromyalgia (FM) is a condition characterised by chronic pain, which may or may not be associated with muscular stiffness, and is commonly accompanied by fatigue, sleep disturbances, and depression. Its aetiopathogenesis cannot be attributed to any specific cause [1]. Prevalence estimates are highly variable (0.4–11%), with a predominance of over 90% in females [2].

Because FM leads to fatigue and a reduction in quality of life, patients often adopt a sedentary lifestyle. One of the main consequences of this lack of physical activity is the loss of muscle mass and strength, corresponding to the definition of sarcopenia [3].

The prevalence of sarcopenia increases proportionally with age. According to various studies, it has been diagnosed in 9.9–40.4% of older adults living in the community [4,5], 2–34% of outpatients [6], and up to 56% of hospitalised patients [7]. These individuals experience greater difficulty in performing activities of daily living, an increased risk of falls and fractures [8], and longer hospital stays [9].

Patients diagnosed with FM often present with muscle weakness; however, this is not always associated with a loss of muscle mass [5]. Such weakness may be due to low levels of physical activity, which are directly related to the widespread pain characteristic of the condition [10].

Regarding body composition, it has been demonstrated that obesity directly influences factors that increase pain perception and disease severity and reduce quality of life [11]. Anthropometric measurements commonly assessed in studies involving FM include height, weight, body mass index (BMI), and circumferences of the waist, trochanter, and dominant thigh [12].

Another variable of importance in patients with FM or sarcopenia is the cross-sectional area (CSA) and thickness of the vastus lateralis of the quadriceps, as this muscle head has the greatest capacity for force generation [13]. These two variables are typically assessed via ultrasound. In a study conducted on older adults, the mean thickness of the vastus lateralis in healthy subjects was reported to be 1.9 cm [14].

The primary objective of this study was to examine the association between FM and the elevated risk of developing sarcopenia according to SARC-F. A secondary objective was to analyse the relationship between these conditions and various aspects of quality of life, as well as with body composition variables.

## 2. Materials and Methods

### 2.1. Study Design and Participants

This was a descriptive observational study. The sample consisted of 84 volunteer patients diagnosed with FM who were members of the AFINSYFACRO Association in Móstoles, Spain, and had received a medical diagnosis of FM from qualified healthcare professionals. Both men and women aged between 18 and 75 years were eligible.

The sample size was calculated using QuestionPro, applying a 95% confidence interval and a 5% margin of error.

Inclusion criteria were (I) confirmed diagnosis of FM; (II) age between 18 and 75 years; (III) voluntary participation with signed informed consent; (IV) no recent surgeries; and (V) adequate cognitive capacity to complete the questionnaires administered during the study.

### 2.2. Variables

Sociodemographic characteristics include age, BMI, marital status, employment status, and presence of comorbidities.

Pain was assessed using the VAS, a validated 0–10 scale for FM patients that measures pain intensity, where 0 represents no pain, and 10 indicates the worst imaginable pain. Test–retest reliability has been shown to be high, particularly among literate patients (r = 0.94; *p* < 0.001) [15].

### 2.3. Anthropometric and Clinical Measurements

Algometry: Pressure pain thresholds were measured using a Fischer analogue algometer (FNP100) at tender points located on the upper limbs (right and left epicondyles) and lower limbs (greater trochanter and medial knee bilaterally) [16].

Circumference measurements: Waist, trochanter, and dominant thigh circumferences were measured using a flexible tape measure.

Muscle architecture measurements: To take the measurements, patients were placed in a supine position with their limbs relaxed and in anatomical position. The anatomical point for measuring muscle architecture variables related to VL was 50% of the distance from the greater trochanter to the knee joint line. The points were marked with a marker pen for probe placement with a neutral inclination and perpendicular to the longitudinal axis of the limb. Conductive gel was applied prior to probe contact. Three images were taken of each measurement, and the mean value of these was used for analysis [17,18,19].

### 2.4. Questionnaires

Sleep quality was assessed using the Pittsburgh Sleep Quality Index (PSQI), which evaluates both qualitative and quantitative aspects of sleep. The questionnaire comprises 24 items (19 self-rated and 5 rated by a bed partner or roommate, if applicable). It yields seven component scores—subjective sleep quality, latency, duration, habitual efficiency, disturbances, use of hypnotics, and daytime dysfunction—each scored from 0 to 3, where 0 indicates no difficulty and 3 indicates severe difficulty. The global score (0–21) classifies respondents as good sleepers (≤5) or poor sleepers (>5) [20].

Fibromyalgia Impact Questionnaire (FIQ) *Fibroi* is a multidimensional instrument assessing functional capacity and quality of life in FM patients, using the validated Spanish version. Scores between 0 and <39 indicate that the impact is slight, between 39 and <59 moderate, and ≥59 severe [21,22,23].

Risk of developing sarcopenia: We used SARC-F which is a self-administered questionnaire that includes five items assessing strength, walking ability, stair climbing, chair rise, and history of falls, with scores from 0 to 10. A score ≥ 4 is predictive of sarcopenia [24,25].

Hospital Anxiety and Depression Scale (HADS) consists of 14 items divided into two subscales—HADS-A (anxiety) and HADS-D (depression)—each containing seven items. Scores are classified as normal (0–7), borderline (8–10), or clinical case (11–21) [26].

### 2.5. Ethical Considerations

This study complies with Spanish Organic Law 7/2021, of 26 May, concerning the protection of personal data for the purposes of prevention, detection, investigation, and prosecution of criminal offences. The research adhered to the ethical principles of the Declaration of Helsinki (2014) regarding medical research involving human subjects.

All participants were informed of their rights to privacy and confidentiality. Written informed consent was obtained from each participant and is held by the corresponding author.

The study was approved by the Ethics Committee of Hospital Clínico San Carlos (Spain) (approval code: 24/745-EC_X) and registered at ClinicalTrials.gov (identifier: NCT06253273).

### 2.6. Statistical Analysis

Data were analysed using SPSS Statistics, version 29.0 (IBM Corp., Armonk, NY, USA). Categorical variables were expressed as percentages, while quantitative variables were presented as mean and standard deviation (X ± SD) or median and range, as required.

The normality of quantitative variables was tested using the Kolmogorov–Smirnov test. Correlations were explored using Spearman’s rho for non-parametric variables and Pearson’s correlation coefficient for parametric variables.

To compare the values of quantitative variables between the categories of qualitative variables, we used either Student’s t or analysis of variance (ANOVA) or Mann–Whitney’s U or Kruskal–Wallis tests according to normality. Associations between qualitative variables were examined using the chi-squared test.

To test whether the associations found in the univariate analysis were independent and to measure their impact, we performed a logistic regression model. As usual, *p* values < 0.05 were considered significant.

## 3. Results

A total of 96.3% of the participants were women, with a mean age of 54.06 ± 9.67 years and a mean body mass index (BMI) of 31.06 ± 3.50 kg/m^2^. Overall, 56.3% of the sample presented a high risk of developing sarcopenia according to SARC-F.

The results indicate a high prevalence of symptoms related to FM, such as pain, sleep quality, anxiety, and depression. The scores obtained from the scales assessing these parameters are summarised in Table 1.

Table 2 summarises the mean and standard deviation values for algometry, circumferential measurements, and ultrasound variables obtained from the participants.

As shown in Table 3, a positive and significant correlation was observed between BMI and thigh circumference (ThCirc) (r = 0.599; *p* < 0.01), indicating that a higher BMI is associated with greater limb girth. Conversely, VAS scores showed significant negative correlations with anxiety (r = −0.458; *p* < 0.01) and with nearly all algometric measures (*p* < 0.05–0.01). This suggests that higher pain perception is associated with lower pressure pain thresholds and greater emotional distress.

As shown in Table 4, FIQ demonstrated a positive and significant correlation with sleep quality (PSQ)I (r = 0.362; *p* < 0.01) and depression (HADS-D) (r = 0.552; *p* < 0.01)**,** indicating that poorer sleep and higher depressive symptoms are linked to a greater perceived disease impact.

Furthermore, the presence of a high risk for developing sarcopenia according to SARC-F was significantly associated with FIQ scores (*p* = 0.002), depression (*p* < 0.001), and body circumference measurements (waist: *p* = 0.025; thigh: *p* = 0.047).

**Figure 1:**
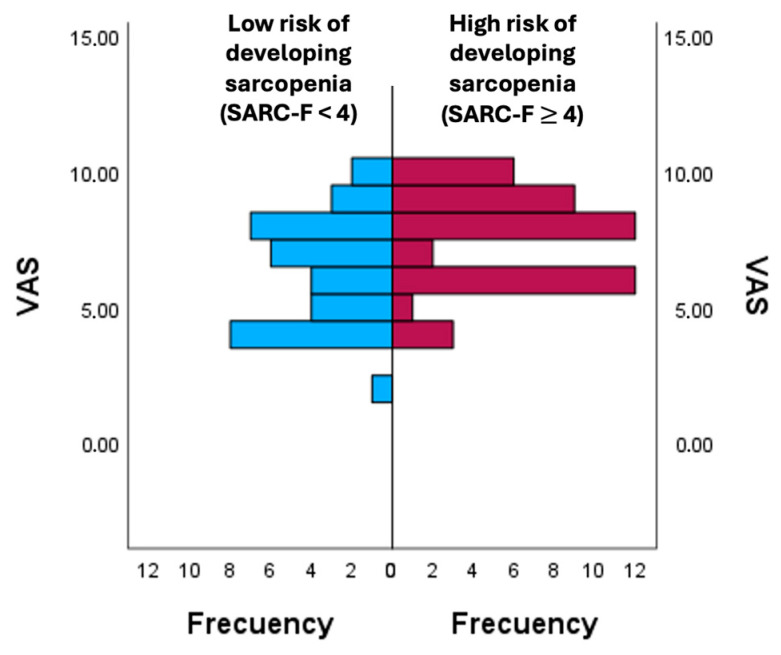
Comparison of pain perception according to the risk of developing sarcopenia. VAS: Visual Analogue Scale.

Figure 1 shows the comparison of pain scores (VAS) between groups stratified according to sarcopenia, the risk of developing sarcopenia according SARC-F. The analysis revealed differences in mean ranks (group with a score < 4 on the SARC-F: 32.86 ± 6.52; group with a score ≥ 4 on the SARC-F: 46.37 ± 7.27), showing higher VAS values in the high-risk group.

**Figure 2:**
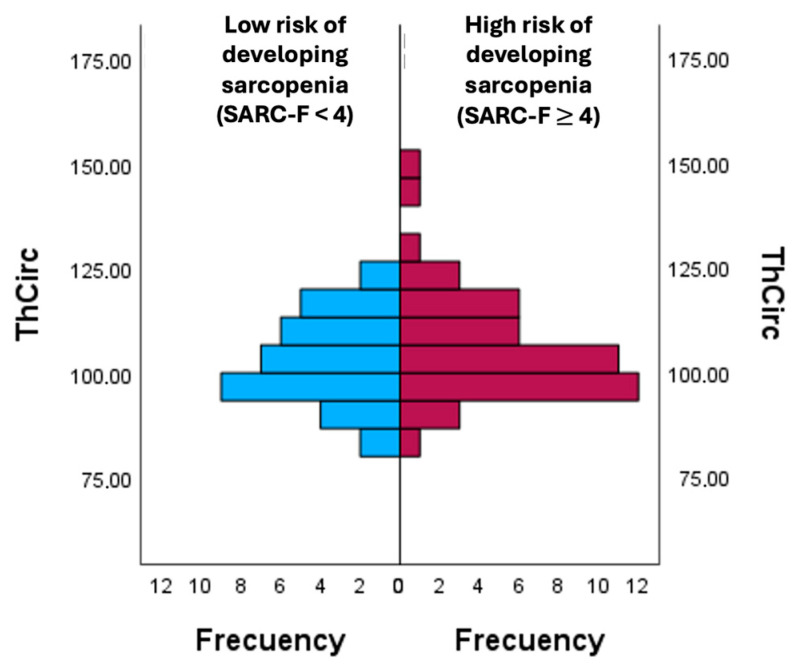
Comparison of ThCirc according to the risk of developing sarcopenia. ThCirc: Trochanter circumference.

In Figure 2, thigh circumference (ThCirc) was compared between participants with the risk of patients developing sarcopenia according to the SARC-F. The group with a score ≥ 4 points on the SARC-F showed a higher mean ThCirc (106.89 ± 13.63) compared to the group with a score < 4 points on the SARC-F (103.06 ± 8.75), reflecting greater ThCirc values among individuals at the higher risk of developing sarcopenia according to the SARC-F.

To show whether the observed effects were independent, we adjusted a logistic regression model for women, as the number of men in our series was too small to allow modelling. The R2 value for the model was 0.51. Results are shown in Table 5.

## 4. Discussion

This study aimed to evaluate the relationship between FM and the risk of developing sarcopenia, as screened by the SARC-F questionnaire, and to examine its associations with pain perception, psychological status, and body composition. Our findings provide evidence supporting an association between FM and a higher risk of developing sarcopenia according to SARC-F, and they are consistent with the notion of a vicious cycle in which chronic pain, reduced physical capacity, and psychosocial burden reinforce each other.

Participants obtained an average score on the FIQ of 65.28 ± 16.08, and 56.3% of the sample presented SARC-F scores ≥ 4. This prevalence is higher than that reported in community-dwelling adults without chronic pain syndromes, suggesting that individuals with FM are more vulnerable to a higher risk of developing sarcopenia [27]. Similar studies have previously reported significant reductions in muscle strength and functional performance in FM patients, contributing to lower health-related quality of life and greater perceived disability [28,29]. The significant correlation observed between the FIQ scores and sarcopenia risk (*p* = 0.002) indicates that the functional and psychosocial burden of FM may be partly mediated by muscle deterioration.

The strong association found between pain intensity and anxiety (r = −0.485; *p* < 0.01) aligns with the well-established interplay between chronic pain, emotional distress, and pain pathways [30]. Evidence suggests that decreased muscle mass and strength are independently associated with higher pain intensity and reduced muscle mass and strength [31]. Moreover, psychological distress can exacerbate muscle catabolism, reinforcing the vicious cycle of pain, weakness, and fatigue.

We observed a reduction in pressure pain thresholds among participants with a high risk of developing sarcopenia, as SARC-F supports the hypothesis that impaired muscle integrity contributes to heightened pain sensitivity. In addition, the current study also confirmed a high prevalence of anxiety and depressive symptoms, consistent with previous reports describing frequent mood disturbances among FM patients and those with sarcopenia [32,33].

Although ultrasound was employed to assess the thickness and CSA of the vastus lateralis—both recognised indicators of muscle mass and quality [34,35,36]—no statistically significant correlation was detected with risk of developing sarcopenia according to SARC-F. This may be attributed to the multifactorial nature of sarcopenia in FM, in which functional impairments can occur even in the absence of measurable muscle atrophy. Inactivity, neuromuscular inefficiency, and altered proprioceptive feedback have all been implicated in the deterioration of motor function in FM, potentially masking the relationship between muscle architecture and clinical risk scores. A study evaluated VL thickness to identify low muscle mass and determined cut-off points of 1.7 cm for men and 1.5 cm for women [37]. Considering that our sample consisted mainly of women and that the average VL thickness was 1.65 cm, this indicates that there was no low muscle mass in these subjects. Anthropometric analyses revealed that waist and thigh circumferences were significantly associated with sarcopenia risk according to SARC-F (*p* = 0.025 and *p* = 0.047, respectively). This finding is consistent with prior research linking central adiposity and unfavourable fat distribution to impaired muscle performance in FM [38,39]. The coexistence of sarcopenic obesity—a phenotype characterised by low muscle mass and high fat accumulation—has been increasingly recognised in FM populations and may exacerbate pain perception, systemic inflammation, and metabolic dysfunction [40,41]. Therefore, the evaluation of body composition parameters should be systematically incorporated into FM management to identify high-risk individuals and guide targeted interventions [42].

Taken together, these multivariate findings complement the bivariate associations previously described, reinforcing the multidimensional nature of risk of developing sarcopenia in fibromyalgia and providing a framework for interpreting the clinical and pathophysiological implications discussed below. To determine the independence of the observed effects, a logistic regression model was fitted for women, given the limited number of men in the sample. The model demonstrated a good fit (R^2^ = 0.51), indicating that the included variables explained more than half of the variability in sarcopenia risk. Among the variables that remained significantly associated were trochanteric algometry (ALGTI and ALGRI) and hormonal status, suggesting that pain sensitivity and hormonal conditions independently contribute to sarcopenia risk prediction. Specifically, lower pressure pain thresholds at trochanteric points were associated with a higher likelihood of sarcopenia, while certain occupational conditions and menopausal status exerted relevant effects within the model. These findings are consistent with previous evidence linking chronic pain and reduced muscle function to neuroendocrine dysregulation and psychosocial stressors in fibromyalgia populations [20,27]. The interplay between hormonal changes, physical inactivity, and heightened pain sensitivity may accelerate muscle deterioration, reinforcing the concept of sarcopenia as a multifactorial condition in fibromyalgia. Clinically, these results underscore the need for comprehensive screening strategies that integrate musculoskeletal assessment with psychosocial and hormonal profiling. Multimodal interventions combining resistance and aerobic exercise, nutritional optimisation, and psychological support have demonstrated efficacy in improving muscle strength, reducing pain sensitivity, and enhancing quality of life in this population [29,30].

The limitations inherent to this study include the omission of the variable of regular physical exercise from the analysis. Neither was it considered whether the patients were taking any pharmacological treatments that could have influenced their physical condition. Furthermore, the sample size was relatively small and exclusively consisted of women, recruitment was limited to one association, and this is a condition that predominantly affects females.

Future longitudinal studies employing objective imaging and biochemical markers are warranted to clarify causal pathways and evaluate the efficacy of multimodal interventions in mitigating sarcopenic progression in FM. Furthermore, a control group of individuals with chronic pain but without FM, or who are healthy, should be included. Understanding these interactions is essential for designing personalised therapeutic strategies aimed at improving functional capacity, psychological well-being, and overall quality of life in affected individuals.

## 5. Conclusions

This study provides evidence of a significant correlation between the impact of FM and an increased risk of developing sarcopenia according to SARC-F. Participants showed high levels of pain, anxiety, and depression along with elevated BMI and circumference measurements, suggesting the coexistence of muscle dysfunction and metabolic imbalance in this population.

The interplay between chronic pain, physical inactivity, and emotional distress appears to promote muscle loss and reduced functional capacity, reinforcing the need to approach FM as a systemic and multidimensional condition.

## Figures and Tables

**Table 1:** Sociodemographic and descriptive variables of the participants.

Variables	Mean ± SD; %
Sex (a/b)	96.3; 3.8
Age	54.06 ± 9.67
Hormonal status(c/d/e/f)	22.5; 20.0; 53.8; 3.8
Smoker (g/h)	20.0; 80.0
Alcohol consumption (i/j/k)	1.3; 36.3; 62.5
BMI (kg/m^2^)	31.06 ± 3.50
FIQ	65.28 ± 16.08
VAS	7.04 ± 1.95
PSQI	14.89 ± 4.29
HADS	12.75 ± 4.55
SARC-F ≥ 4 points	56.3

(a) women. (b) men. (c) I have periods. (d) I don’t have periods. (e) I am menopausal. (f) I am a man. (g) smoker. (h) non-smoker. (i) yes, daily. (j) yes, sometimes. (k) no. BMI: Body Mass Index; FIQ: Fibromyalgia Impact Questionnaire; VAS: Visual Analogue Scale; PSQI: Pittsburgh Sleep Quality Index; HADS: Hospital Anxiety and Depression Scale.

**Table 2:** Values of algometry, circumferential, and ultrasound.

Variables	Mean ± SD
LEAlg (kPa)	1.70 ± 0.71
REAlg (kPa)	170 ± 0.76
GRTAlg (kPa)	1.99 ± 0.93
GLTAlg (kPa)	1.90 ± 0.94
RKAlg (kPa)	1.87 ± 0.92
LKAlg (kPa)	1.88 ± 0.87
ThCirc (cm)	105.69 ± 12.39
TCirc (cm)	53.38 ± 7.32
WCirc (cm)	92.15 ± 13.46
VL Thickness (cm)	1.65 ± 0.31
VL CSA (cm^2^)	11.73 ± 3.77

LEAlg: left epicondyle algometry; REAlg: right epicondyle algometry; GRTAlg: greater right trochanter algometry; GLTAlg: greater left trochanter algometry RKAlg: right knee algometry; LKAlg: left knee algometry; ThCirc: trochanter circumference; TCirc: thigh circumference; WCirc: waist circumference; VL: vastus lateralis and CSA cross-sectional area.

**Table 3:** Correlation between BMI and VAS with anxiety, algometry, and circumferential.

	BMI (kg/m^2^)	VAS	HADS-A (points)	LEAlg (kPa)	REAlg (kPa)	GRTAlg (kPa)	GLTAlg (kPa)	RKAlg (kPa)	LKAlg (kPa)	TCirc (cm)
BMI (kg/m^2^)	1.000	−0.059	0.082	0.182	0.159	−0.126	0.007	−0.082	−0.029	0.599 **
VAS	−0.059	1.000	−0.458 **	−0.409 **	−0.366 **	−0.220	−0.273 *	−0.229 *	−0.249 *	−0.057

BMI: Body Mass Index; VAS: Visual Analogue Scale; HADS: Hospital Anxiety and Depression Scale; LEAlg: left epicondyle algometry; REAlg: right epicondyle algometry; GRTAlg: greater right trochanter algometry; GLTAlg: greater left trochanter algometry RKAlg: right knee algometry; LKAlg: left knee algometry; and TCirc: trochanter circumference. * *p* < 0.005 and ** *p* < 0.001

**Table 4:** Correlation between FIQ, sarcopenia risk, and variables related to age, sleep, depression, circumference, and ultrasound measurements.

	FIQ (Points)	Age	PSQI (Points)	HADS-D (Points)	WCirc (cm)	TCirc (cm)	Thickness VL (cm)	CSA-VL (cm^2^)
FIQ	-	0.042	<0.001	<0.001	0.366	0.190	0.618	0.630
SARC-F ≥ 4 points	0.002	0.416	0.046	<0.001	0.025	0.047	0.143	0.042

FIQ: Fibromyalgia Impact Questionnaire; PSQI: Pittsburgh Sleep Quality Index; HADS: Hospital Anxiety and Depression Scale; WCic: waist circumference; TCirc: thigh circumference; VL: vastus lateralis; and CSAVL: cross-sectional area vastus lateralis.

**Table 5:** Logistic regression model.

Variable	Risk (Exp *β*)	*p*-Value	CI (95%)
PSQI	0.787	0.012	0.652–0.949
HADS-A	1.2	0.06	0.99–1.46
GLTAlg	3.7	0.01	1.25–10.9
LKAlg	3.7	0.04	1-05–13.27
Menstrual state	0.4	0.03	0.17–0.93
Constant	0.013	0.02	-

PSQI: Pittsburgh Sleep Quality Index; CI: confidence interval; HADS: Hospital Anxiety Depression Score; GLTAlg: greater left trochanter; LKAlg: left knee algometry.

## Data Availability

The data present in this study are available upon reasonable request from the corresponding author.

## Abbreviations

The following abbreviations are used in this manuscript:

BMI	Body Mass Index
CSA	Cross-Sectional Area
FIQ	Fibromyalgia Impact Questionnaire
FM	Fibromyalgia
GRTAlg/FLTAlg	Greater Left Trochanter Algometry/Greater Right Trochanter Algometry
HADS	Hospital Anxiety Depression Scale
LEA/REA	Left Epicondyle Algometry/Right Epicondyle Algometry
PSQI	Pittsburgh Sleep Quality Index
RKAlg/LKAlg	Right Knee Algometry/Left Knee Algometry
TCirc	Tight Circumference
ThCirc	Trochanter Circumference
VAS	Visual Analogue Scale
WCirc	Waist Circumference
GLTAlg	Greater Left Trochanter Algometry
RKAlg	Right Knee Algometry
LKAlg	Left Knee Algometry
VL	Vastus Lateralis
